# Morphology and growth of mammalian cells in a liquid/liquid culture system supported with oxygenated perfluorodecalin

**DOI:** 10.1007/s10529-013-1218-2

**Published:** 2013-05-12

**Authors:** Maciej Pilarek, Iwona Grabowska, Maria A. Ciemerych, Katarzyna Dąbkowska, Krzysztof W. Szewczyk

**Affiliations:** 1 Biotechnology and Bioprocess Engineering Division, Faculty of Chemical and Process Engineering, Warsaw University of Technology, Waryńskiego 1, 00-645 Warsaw, Poland; 2 Department of Cytology, Faculty of Biology, Institute of Zoology, University of Warsaw, Miecznikowa 1, 02-096 Warsaw, Poland

**Keywords:** Animal cell culture, Flexible interfacial area, Liquid/liquid culture system, Multiphase bioreactors, Perfluorochemical (perfluorocarbon)

## Abstract

Adherent A431, BHK-21, and C2C12 cells were cultured on a flexible interface formed between two immiscible liquid phases: (i) hydrophobic perfluorodecalin (PFD) and (ii) aqueous culture medium (DMEM). BHK-21 cells formed multicellular aggregates characterized by irregular shapes. A431, as well as C2C12 cells, grew as tight multicellular sheets of 3-D cells. Enhanced mass transfer and facilitated access of the cells to the O_2_ dissolved in PFD/DMEM by approx. 250 % and thereby increased the density of BHK-21 cells. Thus the liquid/liquid system is a simple, ready-to-use, and fully scalable (independent of vessel shapes); consequently it is a method for 3-D cultures of adherent animal cells in which the growth of anchorage-dependent cells is not limited by confluence effect.

## Introduction

The commonly-used systems of adherent animal cells culture require solid surfaces of culture dishes. However, the application of such culture methods is limited by the cells grown under such conditions usually forming surface-attached monolayers (Ulloa-Montoya 2005; Thomson et al. 2007). However, the cells might also form multilayer colonies or aggregates. In such case, the O_2_ supply to every cell might be a limiting factor (Fassnacht and Portner 1999; Harrison et al. 2007). Thus, the application of O_2_ rich fluids, i.e. hydrophobic liquid O_2_ carriers, might be an alternative to conventional aeration systems (Leung et al. 1997; Radisic et al. 2006).

Synthetic liquid perfluorochemicals (PFCs), which dissolve gases according to Henry’s Law, can be used as carriers of O_2_. Moreover, the gas transfer rate into PFCs increases linearly with the partial pressure of a component in the gaseous phase (Riess 2006; Sobieszuk and Pilarek 2012) in contrast to the sigmoid dissociation curve which is characteristic for biological O_2_ carriers. The O_2_ solubility in PFCs is approx. 20 times higher than in water and it does not vary significantly with temperature (Costa et al. 2004). Importantly, liquid PFCs are immiscible with aqueous media and they create a separate liquid lying below the medium, at the bottom of a culture dish. The lack of chemical bonds between O_2_ and PFC also allows the efficient release of O_2_ into the aqueous phase. The lack of toxicity and negative side effects of liquid PFCs on living cells has been confirmed by in vitro experiments and also in clinical investigations (Mattiasson and Adlercreutz 1987; King et al. 1989; Krafft 2001; Lowe 2002; Pilarek and Szewczyk 2008; Castro and Briceno 2010; Pilarek et al. 2012; Hillig et al. 2013).

An innovative bioengineering application of PFCs is the liquid/liquid culture system for in vitro cultures of 3-D aggregated animal cells. Such aggregates can be grown on the liquid/liquid interface created between the hydrophobic PFC and the aqueous culture medium. Data on animal cell cultures at PFC/medium surface are limited and do not provide a detailed analysis of cell morphology, growth characteristics and physiology (Shiba et al. 1998; Rappaport 2003). Thus, the aim of our work was to study mammalian cells cultured at the interfacial area between PFC and aqueous medium. To achieve this, three anchorage-dependent cell types which differ in morphology (epithelial A431 cells, BHK-21 fibroblasts, and C2C12 myoblasts) and in their culture requirements (A431 and C2C12 have a higher anchorage-dependency than BHK-21 cells) have been compared.

## Materials and methods

### Liquid perfluorinated phase

Perfluorodecalin (PFD; C_10_F_18_; ABCR GmbH, Germany) was used as the carrier of respiratory gases. PFD was sterilized by autoclaving, cooled to 37 °C, and filtered using membrane filters (0.2 μm) to remove any solid contamination. Then PFD was saturated by compressed air or pure O_2_ (see Table 1 for detailed concentrations of O_2_) under aseptic conditions (Pilarek and Szewczyk 2008). A culture system, which was not supplemented with PFD, was used as the reference.

**Table 1:** O_2_ concentration in PFD used in the experiments

	Composition of PFD mixture (per ml)	Concentration of O_2_ (μM O_2_ ml^−1^ PFD)		
		From air	From pure O_2_	Total
PFD_(air)_	1 ml PFD_(air)_	4	0	4
${\text{PFD}}_{{( + 20 \% {\text{O}}_{ 2} )}}$	0.8 ml PFD_(air)_ + 0.2 ml ${\text{PFD}}_{{({\text{O}}_{ 2} )}}$	3	4	7
${\text{PFD}}_{{( + 40 \% {\text{O}}_{ 2} )}}$	0.6 ml PFD_(air)_ + 0.4 ml ${\text{PFD}}_{{({\text{O}}_{ 2} )}}$	2	8	10
${\text{PFD}}_{{( + 60 \% {\text{O}}_{ 2} )}}$	0.4 ml PFD_(air)_ + 0.6 ml ${\text{PFD}}_{{({\text{O}}_{ 2} )}}$	2	12	14
${\text{PFD}}_{{( + 80 \% {\text{O}}_{ 2} )}}$	0.2 ml PFD_(air)_ + 0.8 ml ${\text{PFD}}_{{({\text{O}}_{ 2} )}}$	1	15	16
${\text{PFD}}_{{({\text{O}}_{ 2} )}}$	1 ml ${\text{PFD}}_{{({\text{O}}_{ 2} )}}$	0	19	19

*PFD*_(air)_ PFD saturated by atmospheric air, $PFD_{{(O_{2} )}}$ PFD saturated by pure O_2_

PFD_(air)_ and ${\text{PFD}}_{{({\text{O}}_{ 2} )}}$ were mixed to obtain O_2_-enriched PFD

### Animal cells and culture medium

Three anchorage-dependent cell lines were studied: human A431 cell line derived from epidermal carcinoma, mouse C2C12 myoblasts, and hamster BHK-21 fibroblasts. All cells were cultured in Dulbecco’s modified Eagle’s medium (DMEM) supplemented with 10 % (v/v) fetal calf serum, antibiotics (0.05 U penicillin ml^−1^, 0.05 U streptomycin ml^−1^), and 10 mM HEPES at 37 °C. The inoculum of each cell line was prepared from standard, 75–80 % confluent cultures and cells were suspended in DMEM to give 0.5 × 10^5^ cells ml^−1^.

### Experimental procedures

All cells were cultured in closed, sealed 24-well plates (24-WP). Two ml PFD and two ml DMEM were used to the create PFD/medium interface. Cells were cultured using PFD with different amounts of O_2_ (see Table 1), i.e. air-saturated PFD or air-saturated PFD enriched with pure O_2_. There were also two reference systems: (i) cells cultured in PFD/DMEM system with degasified PFD and (ii) cells cultured on solid surface without any PFD.

### Analytical methods

#### Imaging cells

Cells and aggregates were monitored and documented with a inverted light microscope supported with digital camera and Nikon CoolView software. Cell viability, density, and morphology were analyzed every 24 h for 7 days.

#### Counting cells

Cells cultured referentially on solid surface were detached by 3 min incubation in 0.25 % trypsin at 37 °C, and then suspended in 1 ml DMEM. For cultures at the PFD/DMEM interface, almost all DMEM and PFD was removed under vacuum followed by the addition of 1 ml fresh DMEM into the culture. The mixtures were repeatedly pipetted to obtain homogenous suspension of cells in DMEM. Viability of cells was analyzed using the Trypan Blue staining method. Five independent cultures were used to calculate the mean value of living cell densities for every time point of cell culture.

#### Specific glucose consumption rate measurement

The metabolic activity of cells was estimated by measurement of the specific glucose consumption rate (*r*_*glc/cells*_) using following equation:

$$ r_{glc/cells} = - \frac{{\Updelta C_{glc} }}{\Updelta t \cdot l} ( {\text{mg}}\,{\text{ml}}^{ - 1} \,{\text{h}}^{ - 1} \,{\text{cells}}^{ - 1} ) $$

where *ΔC*_*glc*_ is glucose concentration change between two points, *Δt* = time between two points and *l* = cells/ml. Glucose was measured by capillary zone electrophoresis technique with an Agilent CE-3D system equipped with a diode array detector and 485 mm capillary (50 μm inner diameter) (Soga and Serwe 2000). 20 mM 2,6-Pyridinedicarboxylic acid and 0.5 mM cetyltrimethylammonium bromide was applied as separation buffer (pH 12.1). Peak of glucose was indirectly detected at λ = 300 nm (with λ = 275 nm as reference). An Agilent ChemStation software was used for data integration and the internal standard method was applied for the quantitative analysis of glucose in culture medium samples directly.

## Results and discussion

### Liquid/liquid culture system

**Fig. 1:**
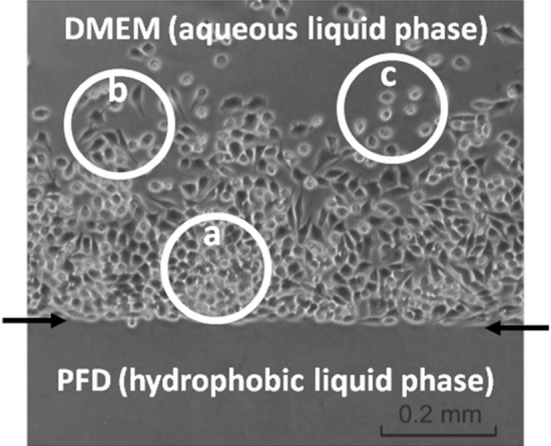
Growth of the BHK-21 cells in PFD/DMEM culture system: cells closely packed in 3-D multicellular aggregate (**a**), elongated cells attached to side-surface of culture flask (**b**), and single spherical cells freely floated in aqueous phase of DMEM (**c**). Interfacial area of hydrophobic PFD and aqueous phase of culture medium has been marked with *arrows*

An example of the flexible liquid/liquid culture system containing the air-saturated liquid PFD and DMEM has been presented on Fig. 1. The BHK-21 cells localized at the interfacial area of two immiscible liquid phases, at the hydrophilic side of the interface. Cells were absent from the hydrophobic side of interfacial area. Some cells grew on the culture dish walls in the immediate vicinity of the PFC/DMEM interfacial area of. The number of cells localized at the walls decreased with the distance from the interface. The cells located at the PFD/DMEM interface and walls of culture dish were counted separately. Fewer than 1 % of cells grew on the walls and were therefore disregarded. The BHK-21 cells which grew at the flexible PFD/DMEM interface had a 3-D morphology and were closely packed in 3-D multicellular aggregates (Fig. 1). The liquid/liquid culture system presented here can be used in culture vessel of any shape (e.g. dish/flask, multi-well plate etc.) and is also fully scalable.

### Morphology of cells cultured on PFD/DMEM interface

**Fig. 2:**
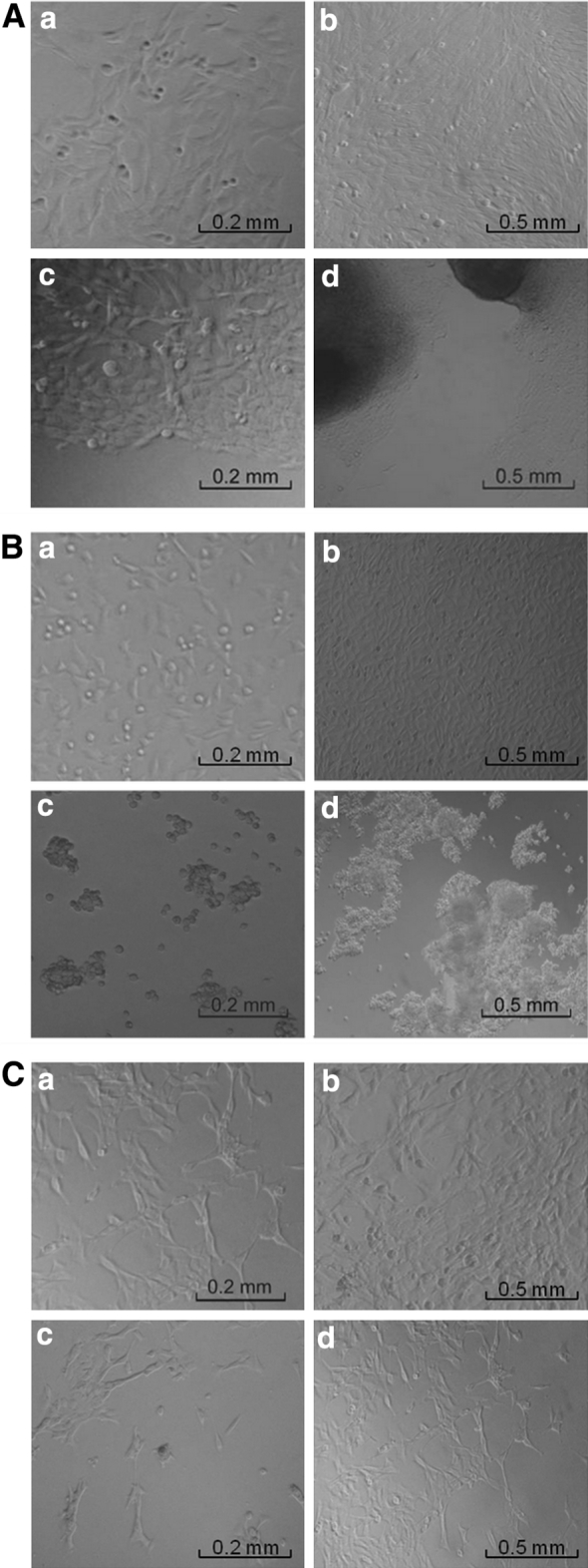
The comparison of the cells morphology of A431 (**A**), BHK-21 (**B**) and C2C12 (**C**) cells cultured on the solid surface (i.e. the control cultures): 2nd (*a*) and 4th (*b*) day of culture; and those ones cultured on the PFD/DMEM interface: 2nd (*c*) and 4th (*d*) day of culture. *Scale bar* = 0.2 mm

A431, BHK-21 and C2C12 cells adhered to the solid surface of 24-WP after 5-6 h. After 4–5 days, all cells formed a confluent monolayer (Fig. 2Ab, Bb, Cb).

A431 cells cultured in liquid/liquid system adhered to the PFD/DMEM interface within 24 h of the adaptation phase. Gentle mixing of culture medium, caused by pipetting of small amount of liquid (DMEM or PFD) or by gentle tapping the culture flask/plate, did not influence detachment or movement of the cells. After 5 days of culture the distinct multilayered aggregates of the A431 cells were visible at the PFD/DMEM interface (Fig. 2Ad).

BHK-21 cells did not adhere to the surface of PFD (Fig. 2Bc, Bd), and they remained spherical for as long as 7 days of culture. Multicellular aggregates of BHK-21 cells were formed within 24-48 h of culture (Fig. 2Bc) and they floated if culture medium was pipetted or the plate was shaken. This showed that adherent BHK-21 cells in the hydrophobic/aqueous two liquid phase culture system grew without being attached to the hydrophobic interfacial area. As a result, harvesting cells in order to analyze their viability and growth was greatly facilitated and the enzyme-mediated detachment of aggregated BHK-21 cells was unnecessary. Consequently, BHK-21 cells grew in PFD/DMEM system could be classified as a 3-D cell culture.

C2C12 cells were cultured under conditions supporting their proliferation (Grabowska et al. 2011). However, after 7 days C2C12 cells did not spread on all accessible PFD/DMEM interfacial area and grew in “grid-like” form (Fig. 2Cd). The adhesion of C2C12 cells to PFD surface seemed to be weak because the pipetting of DMEM triggered the detachment of cells from the interfacial area.

The results of the analyzes of A431, BHK-21, and C2C12 cells presented in the current work, compared to those of L-929 and HepG2 cells published by Shiba et al. (1998) and by Rappaport (2003), confirmed that not all types of cells adapted to the culture conditions of the liquid/liquid culture system in the same way. It seems that cells with lower anchorage-dependency (e.g. BHK-21 and L-929 cells) adapt more effectively to the conditions of flexible interface area between PFC and culture medium than cells characterized by the higher affinity to the solid surface (e.g. C2C12 myoblasts).

**Fig. 3:**
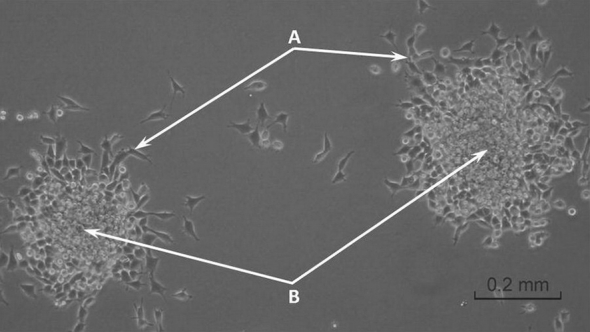
Morphology of the BHK-21 cells forming 3-D aggregates in PFD/DMEM culture system after their subcultivation onto the solid surface (12 h after transfer): the typically elongated fibroblasts migrated from the multicellular aggregates and adhered to the bottom of culture flask (*A*) and the cells still closely packed inside the 3-D aggregates (*B*). *Scale bar* = 0.2 mm

To document the fact that changes in the morphology of cells forming aggregates in PFD/DMEM system are fully reversible, we checked on the ability of aggregate-forming cells to grow at the solid surfaces. The multicellular aggregates of BHK-21 cells were passaged from PFD/DMEM interface directly onto solid surface of 24-WP. Analysis performed after next 24 h revealed that BHK-21 cells positioned in the middle of aggregates retained their 3-D morphology. Whereas some of the cells located near the edge of aggregates became flatten, elongated and started to adhere to solid surface (Fig. 3). Such reversibility in the morphology of 3-D aggregated cells cultured on PFC layer has never been previously described in the literature before.

### The quantitative analysis of growth and viability of cells cultured in the PFD/DMEM culture system

**Fig. 4:**
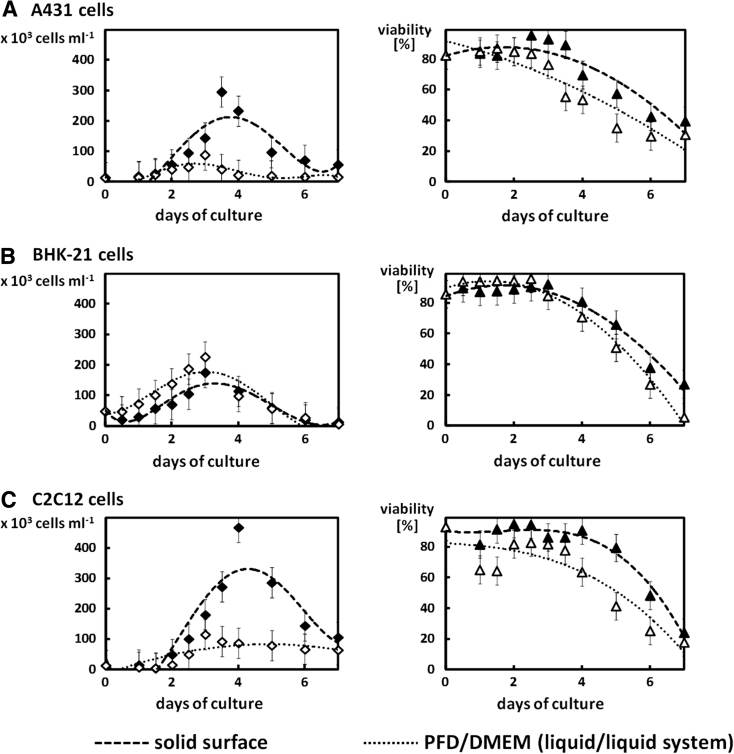
Comparison of growth curves and the viability of A431 (**a**), BHK-21 (**b**), and C2C12 (**c**) cells cultured on the solid surface (i.e. the control cultures) and at the PFD/DMEM interface

The number of cells was higher when they were cultured at the flexible PFD/DMEM interface only in the case of BHK-21 cells (Fig. 4). The differences observed between the growth curves could probably have resulted from the strong anchorage-dependency of A431 and C2C12 cells, which has been repeatedly described (Milasincic et al. 1996; Atsumi et al. 2008). Despite the cells adhering to the flexible interfacial area between two liquid phases (see Fig. 2A, C), they require a solid surface to attach and proliferate (see Fig. 4A, C).

**Fig. 5:**
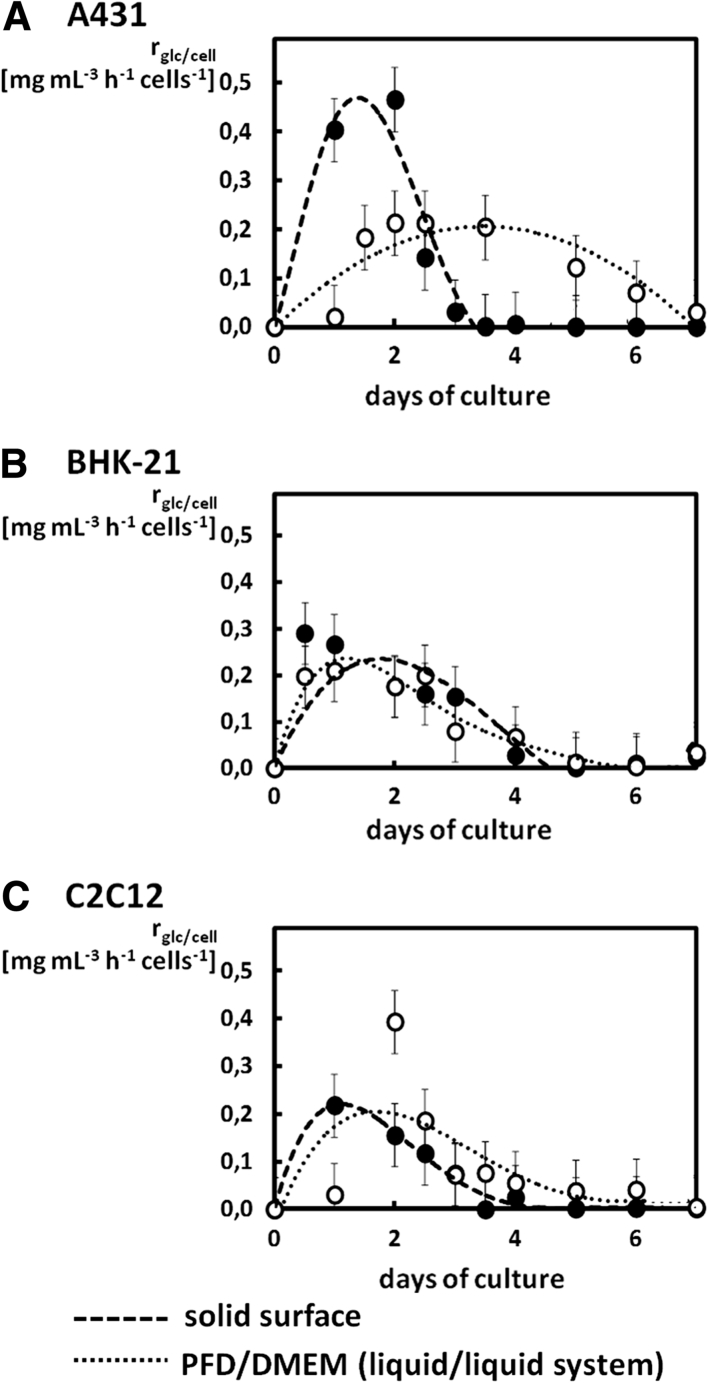
Comparison of the specific glucose consumption rate estimated for A431 (**a**), BHK-21 (**b**), and C2C12 (**c**) cells cultures on the solid surface (i.e. the control cultures) and on the flexible PFD/DMEM interface

To show how the conditions of the PFD/DMEM culture system influenced the metabolic activity of cultured cells, we have analyzed specific glucose consumption rates for A431, BHK-21 and C2C12 cells (Fig. 5). High rates of glucose consumption occurred during the first 3 days for BHK-21 and C2C12 cells in PFD/DMEM system and may be the result of moderate specificity of BHK-21 and C2C12 cells to grow at the solid surface. The rather small values of the specific glucose consumption rate observed for A431 cells cultured at the PFD/DMEM interface clearly indicated problems with their adaptation to the conditions of the liquid/liquid system.

### The effect of O_2_-enriched PFD on the growth of BHK-21 cells

**Fig. 6:**
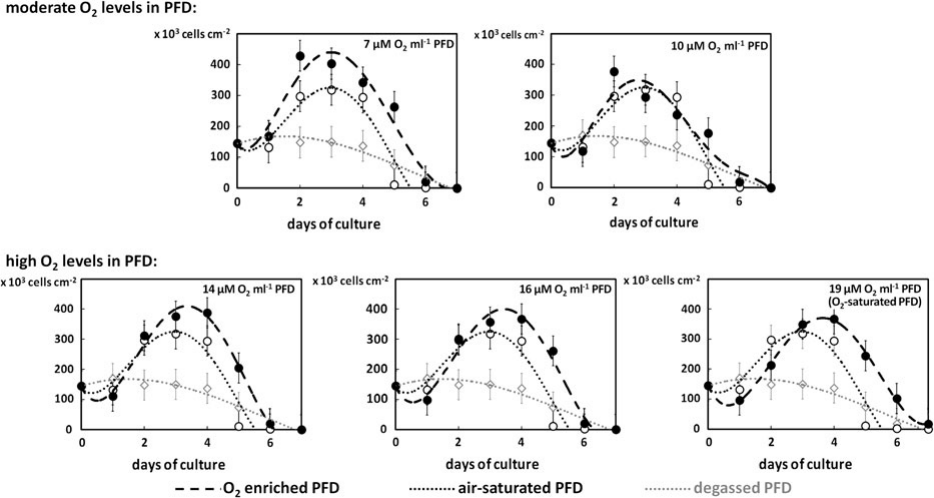
Growth curves of the BHK-21 cells cultured in PFD/DMEM culture system with O_2_-enriched phase of PFD (*black*) compared to control cultures with degassed and air-saturated PFD (*grey*)

The BHK-21 cells were chosen to analyze the effect of O_2_ level in the PFD/DMEM culture system (Fig. 6) because these cells were characterized by the best adaptation to such culture conditions as compared to A431 or C2C12 cells. We decided to start our culture plating 3 times more cells than in previous experiments (1.5 × 10^5^ cells ml^−1^ as compared to 0.5 × 10^5^ cells ml^−1^). This allowed us to check the usefulness of our system in the case of higher cell density cultures. The culture systems containing air-saturated PFD and degassed PFD (i.e. not saturated with any gas) were used as references.

Based on the results presented in Fig. 6, a correlation between the level of O_2_ in PFD and the proliferation rate of the BHK-21 cells could be clearly seen. In the case of the cultures with O_2_ concentrations exceeding normal levels, the maximum in the growth curve was observed at more advanced stages of culture. Simultaneously, higher O_2_ concentration resulted in the decrease in the density of BHK-21 cells cultured in PFD/DMEM culture system. Furthermore, the initial drop in the cell number was observed at the growth adaptation phase in the case of all cultures but it was more significant in cultures using highly O_2_-enriched PFD.

Generally, the growth of mammalian cells is limited where there are higher O_2_ levels in the culture medium compared to systems equilibrated with air. However, we noted that the BHK-21 cells proliferated intensively when exposed to O_2_-enriched PFD. Robust growth of cells in cultures enriched with O_2_ has been reported for mouse hybridoma cells (Wang et al. 1994) and also for TK6 and Vero cells (Oller et al. 1989).

The increased growth rate of BHK-21 cells was characteristic for PFD/DMEM system in that PDF was moderately enriched with O_2_ (7 μM O_2_ ml^−1^) and might result from an enhanced and facilitated access of actively metabolizing cells to O_2_. Thus, we hypothesize that using such a method could have significant consequences for bioprocess control in the propagation of animal cell biomass or for cell-derived bioproducts (such as hormones, erythropoietin, monoclonal antibodies, etc.). As a result of this, O_2_ transfer which is one of the limiting factors in high-cell density cultures of animal cells could be excluded.

## Conclusion

The proposed liquid/liquid culture system may have promising applications in any animal cell cultures whereby the 3-D structure of cells/aggregates should be retained. This flexible (independent of vessel shape) system is simple and ready-to-use and does not require any scaffolds or inserts traditionally used for 3-D cultures of animal cells. It also does not need any sophisticated modification, coating or supporting of the liquid/liquid interfacial area. Robust growth of 3-D aggregated mammalian adherent cells (e.g. BHK-21 cells) was achieved in the liquid/liquid culture system. Under such conditions, the growth of cells was not limited by the available area or by confluence as is observed in traditional solid-surface-based culture systems. Other benefit of the PFC/medium system is its availability to be used for testing of cell growth in the presence of any gaseous compounds, for example it could be applied as a simple device in hypoxia or hyperoxia of animal cells/tissues in vitro studies.
